# Ubiquitin C-Terminal Hydrolase-L1 (UCH-L1) in Prediction of Computed Tomography Findings in Traumatic Brain Injury; a Meta-Analysis

**Published:** 2018-12-15

**Authors:** Fatemeh Ramezani, Amir Bahrami-Amiri, Asrin Babahajian, kavous Shahsavari Nia, Mahmoud Yousefifard

**Affiliations:** 1 Physiology Research Center, Faculty of Medicine, Iran University of Medical Sciences, Tehran, Iran; 2 Occupational Medicine Research Center, Iran University of Medical Sciences, Tehran, Iran; 3 Liver and Digestive Research Center, Kurdistan University of Medical Sciences, Sanandaj, Iran; 4 Road Traffic Injury Research Center, Tabriz University of Medical Sciences, Tabriz, Iran.

**Keywords:** Ubiquitin C-terminal Hydrolase-L1, Traumatic Brain injuries, Diagnosis, Brain computed tomography

## Abstract

**Introduction:**

Ubiquitin C-terminal hydrolase-L1 (UCH-L1) is one of the promising candidates, with an acceptable diagnostic value for predicting head computed tomography (CT) scan findings. However, there has been a controversy between studies and still, there is no general overview on this. Therefore, the current systematic review and meta-analysis attempted to estimate the value of UCH-L1 in predicting intracranial lesions in traumatic brain injury.

**Methods:**

Two independent reviewers screened records from the search of four databases Medline, Embase, Scopus and Web of Science. The data were analyzed in the STATA 14.0 statistical program and the findings were reported as a standardized mean difference (SMD), summary receiver performance characteristics curve (SROC), sensitivity, specificity, and diagnostic odds ratio with 95% confidence interval (95% CI).

**Results:**

Finally, the data of 13 articles were entered into the meta-analysis. The mean serum level of UCH-L1 was significantly higher in patients with CT-positive than in TBI patients with CT negative (SMD = 1.67, 95% CI: 1.12 to 2.23, I2 = 98.1%; p <0.0001). The area under the SROC curve for UCH-L1 in the prediction of intracranial lesions after mild TBI was 0.83 (95% CI: 0.80 to 0.86). Sensitivity, specificity and diagnostic odds ratio of serum UCH-L1 was 0.97 (95% CI: 0.92 to 0.99), 0.40 (95% CI: 0.30 to 0.51) and 19.37 (95% CI: 7.25 to 51.75), respectively. When the analysis was limited to assessing the serum level of UCH-L1 within the first 6 hours after mild TBI, its sensitivity and specificity increased to 0.99 (95% CI: 0.94 to 1.0) and 0.44 (95% CI: 0.38 to 0.052), respectively. In addition, the diagnostic odds ratio of 6-hour serum level of UCH-L1 in the prediction of intracranial lesions was 680.87 (95% CI: 50.50 to 9197.97).

**Conclusion:**

Moderate level of evidence suggests that serum/plasma levels of UCH-L1 have good value in prediction of head CT findings. It was also found that evaluation of serum/plasma level of UCH-L1 within the first 6 hours following TBI would increase its predictive value. However, there is a controversy about the best cutoffs of the UCH-L1.

## Introduction

Traumatic brain injury (TBI) is one of the most common causes of death and disability with a global prevalence of 8.4%. Incidence, prevalence, and years of life lost due to TBI increased significantly from 1990 to 2016 (1). This increasing burden has led to a substantial increase in the TBI-related emergency visits.

Intracranial lesions caused by TBI are diagnosed mainly with imaging assessments such as computed tomography (CT) scan and magnetic resonance imaging (MRI). However, for two reasons, the use of imaging techniques in emergency departments is not feasible. First, there is no access to CT scan and MRI in all emergency departments, and secondly, Imaging is not possible for all trauma patients. In addition, exposure to ionizing radiation as a result of CT scan should not ignore (2, 3). Therefore, researchers are looking for other diagnostic or screening modalities that can be used to detect the intracranial lesion.

Patients with TBI are divided into three categories of mild, moderate and severe, based on clinical evaluations, in particular, Glasgow coma scale (GCS). Mild TBI is the most prevalent and physicians are always in trouble when patients need to go for radiography for further evaluation. Estimates have shown that about 16% of mild TBI patients have intracranial lesions (4). This means that if all mild TBI patients undergo CT scan or MRI, 84% of these imaging is unnecessary. To reduce this excessive imaging, screening tests such as serum biomarkers can be effective.

Several biomarkers such as S100-B and neuron-specific enolase have been proposed to predict central nervous system injuries (5-7), but there is still a controversy between literature, and researchers are still looking for other biomarkers. Of these, ubiquitin C-terminal hydrolase-L1 (UCH-L1) is one of the promising candidates with an acceptable value in the prediction of intracranial lesions (8). However, there has been a contention between studies and there is still no general overview of this area. Therefore, the present systematic review and meta-analysis attempted to evaluate the value of UCH-L1 in the prediction of intracranial lesions in TBI patients.

## Methods

**Search strategy**

The present meta-analysis was designed to determine the value of UCH-L1 in predicting of head CT findings. For this purpose, an extensive search was performed in Medline (via PubMed), Embase, Scopus, and Web of Science. The search terms were related to TBI in combination with the UCH-L1. The Medline search query is provided below. In addition, the search was done manually in the bibliography of relevant studies and review articles. Google's search engine was also searched for gray literature. 

**PubMed search query:**

1- "Ubiquitin Thiolesterase"[mh] OR "Thiolesterase, Ubiquitin"[tiab] OR "Ubiquitin C-Terminal Hydrolase"[tiab] OR "C-Terminal Hydrolase, Ubiquitin"[tiab] OR "Hydrolase, Ubiquitin C-Terminal"[tiab] OR "Ubiquitin C Terminal Hydrolase"[tiab] OR "Ubiquitin Carboxy-Terminal Hydrolase"[tiab] OR "Carboxy-Terminal Hydrolase, Ubiquitin"[tiab] OR "Hydrolase, Ubiquitin Carboxy-Terminal"[tiab] OR "Ubiquitin Carboxy Terminal Hydrolase"[tiab] OR "Ubiquitin C-Terminal Esterase"[tiab] OR "C-Terminal Esterase, Ubiquitin"[tiab] OR "Esterase, Ubiquitin C-Terminal"[tiab] OR "Ubiquitin C Terminal Esterase"[tiab] OR "Ubiquitin Carboxy-Terminal Esterase"[tiab] OR "Carboxy-Terminal Esterase, Ubiquitin"[tiab] OR "Esterase, Ubiquitin Carboxy-Terminal"[tiab] OR "Ubiquitin Carboxy Terminal Esterase"[tiab] OR "Ubiquitin Carboxyl-Terminal Hydrolase Isozyme L1"[tiab] OR "Ubiquitin Carboxyl Terminal Hydrolase Isozyme L1"[tiab] OR "Parkinson Disease 5 Protein"[tiab] OR "PARK5 Protein"[tiab] OR "Neuron Cytoplasmic Protein 9.5"[tiab] OR "UCHL1 Protein"[tiab] OR "Uch-L1 Protein"[tiab] OR "Uch L1 Protein"[tiab] OR "UCH-L1"[tiab]

2- "Brain Concussion"[mh] OR "Brain Injuries"[mh] OR "Brain Injuries, Traumatic"[mh] OR "Brain Concussion"[tiab] OR "Brain Injuries"[tiab] OR "Brain Injuries, Traumatic"[tiab] OR "Brain Concussions"[tiab] OR "Concussion, Brain"[tiab] OR "Commotio Cerebri"[tiab] OR "Cerebral Concussion"[tiab] OR "Cerebral Concussions"[tiab] OR "Concussion, Cerebral"[tiab] OR "Concussion, Intermediate"[tiab] OR "Intermediate Concussion"[tiab] OR "Intermediate Concussions"[tiab] OR "Concussion, Severe"[tiab] OR "Severe Concussion"[tiab] OR "Severe Concussions"[tiab] OR "Concussion, Mild"[tiab] OR "Mild Concussion"[tiab] OR "Mild Concussions"[tiab] OR "Mild Traumatic Brain Injury"[tiab] OR "Injuries, Brain"[tiab] OR "Brain Injury"[tiab] OR "Injury, Brain"[tiab] OR "Injuries, Acute Brain"[tiab] OR "Acute Brain Injuries"[tiab] OR "Acute Brain Injury"[tiab] OR "Brain Injury, Acute"[tiab] OR "Injury, Acute Brain"[tiab] OR "Brain Injuries, Acute"[tiab] OR "Brain Lacerations"[tiab] OR "Brain Laceration"[tiab] OR "Laceration, Brain"[tiab] OR "Lacerations, Brain"[tiab] OR "Brain Injuries, Focal"[tiab] OR "Brain Injury, Focal"[tiab] OR "Focal Brain Injury"[tiab] OR "Injuries, Focal Brain"[tiab] OR "Injury, Focal Brain"[tiab] OR "Focal Brain Injuries"[tiab] OR "Brain Injury, Traumatic"[tiab] OR "Traumatic Brain Injuries"[tiab] OR "Trauma, Brain"[tiab] OR "Brain Trauma"[tiab] OR "Brain Traumas"[tiab] OR "Traumas, Brain"[tiab] OR "TBI (Traumatic Brain Injury)"[tiab] OR "Encephalopathy, Traumatic"[tiab] OR "Encephalopathies, Traumatic"[tiab] OR "Traumatic Encephalopathies"[tiab] OR "Injury, Brain, Traumatic"[tiab] OR "Traumatic Encephalopathy"[tiab] OR "TBIs (Traumatic Brain Injuries)"[tiab] OR "TBI (Traumatic Brain Injuries)"[tiab] OR "Traumatic Brain Injury"[tiab] OR "TBI"[tiab]

3- #1 AND #2

**Selection criteria**

All of the observational studies on the predictive value of UCH-L1 in the prediction of head CT findings were included. Exclusion criteria were chronic exposure to head trauma, penetrating TBI, non-traumatic injury, lack of data, and reviews.

**Data extraction and quality assessment**

The method of summarizing data has been reported in our previous meta-analyses study (9-23). Two independent reviewers screened records from the database. The potentially relevant studies were assessed in detail and finally, based on the selection criteria eligible studies were identified. The reviewers recorded the type of study (cohort, case-control, cross-sectional, etc.), the age range of patients, sample size (number of CT positive and CT negative TBI patients), male gender frequency, sampling method (random, consecutive, convenience), the method of UCH-L1 assay, TBI severity and outcomes. The severity of TBI was divided into three groups, mild (GCS: 13 to 15), moderate (GCS: 9 to 12) and severe (GCS: 3 to 8). The evaluated outcomes included the mean serum level of UCH-L1 in both CT positive and CT negative groups and the number of true positive (TB), true negative (TN), false positive (FP) and false negative (FN).

In some articles, the mean serum level of UCH-L1 was reported in the graphs. In these cases, using the Plot Digitizer software (available at http://plotdigitizer.sourceforge.net/), the mean and standard deviation of the serum level of this biomarker were extracted. In addition, many articles were not reported TP, TN, FP, and FN cases. Therefore, TP, TN, FP, and FN were estimated using the reported sensitivity and specificity.

Quality control of the eligible studies was evaluated using the proposed method of Quality Assessment of Diagnostic Accuracy Studies 2 (QUADAS-2) guideline (24).

**Statistical Methods**

Data were analyzed in the STATA 14.2 program. Meta-analysis was performed in two sections. In the first part, the mean serum level of UCH-L1 was compared in CT positive and CT negative groups. In this section, the standardized mean difference (SMD) was calculated and finally, an overall SMD with a 95% confidence interval (95% CI) was reported. In the second section, using the TP, TN, FP, and FN data, the summary receiver operating characteristic curve (SROC), sensitivity, specificity, diagnostic score, and diagnostic odds ratio of UCH-L1 in the prediction of CT findings were reported. I^2^ test was used to assess heterogeneity and Eager's test was used to evaluate the publication bias. In all analyzes p <0.05 was defined as significant level.

## Results:

***Study characteristics***

**Figure 1:**
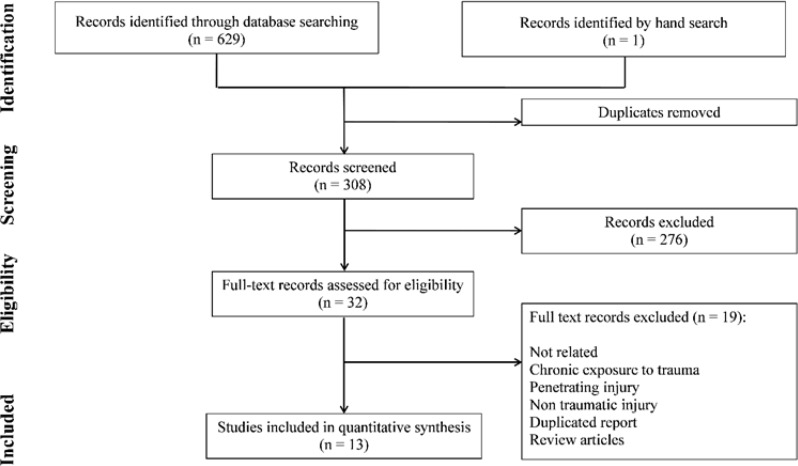
PRISMA flow diagram of present meta-analysis

Finally, 13 articles provided data suitable for meta-analysis (Figure 1) (8, 25-36). They were 12 cohort studies and 1 observational trial. These studies included 3977 patients with TBI. CT scan findings in 3179 (79.93%) patients were negative and in 798 (20.07%) were positive. The serum sample was obtained in five studies during the first 6 hours after the onset of TBI. In two studies, it was assessed within 12 hours and in six studies over the first 24 hours after TBI. Four studies were conducted on mild TBI, five with mild to moderate TBI and four with mild to severe TBI patients. Table 1 shows a summary of the eligible studies.

***Quality control and risk of bias***

**Figure 2:**
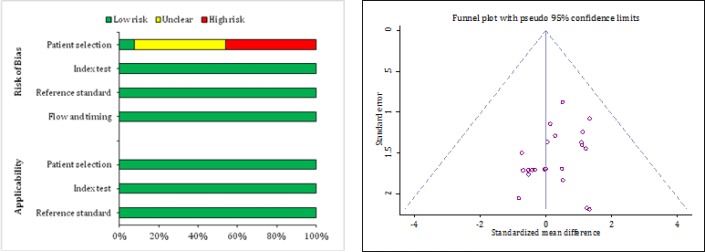
Assessment of risk of bias, applicability (A) and publication bias (B) among eligible studies. No publication bias was observed (p=0.362).

Quality assessment of the relevant studies according to the QUADAS-2 guidelines showed that the risk of bias in patient selection was high or unclear in 12 studies. Other items in all studies were rated as the Low risk of bias (Figure 2A and Table 2). There was no publication bias in the present study (p = 0.362) (Figure 2B).

**Meta-analysis**

**Comparison of mean serum/plasma UCH-L1 in CT positive and CT negative patients**

**Figure 3:**
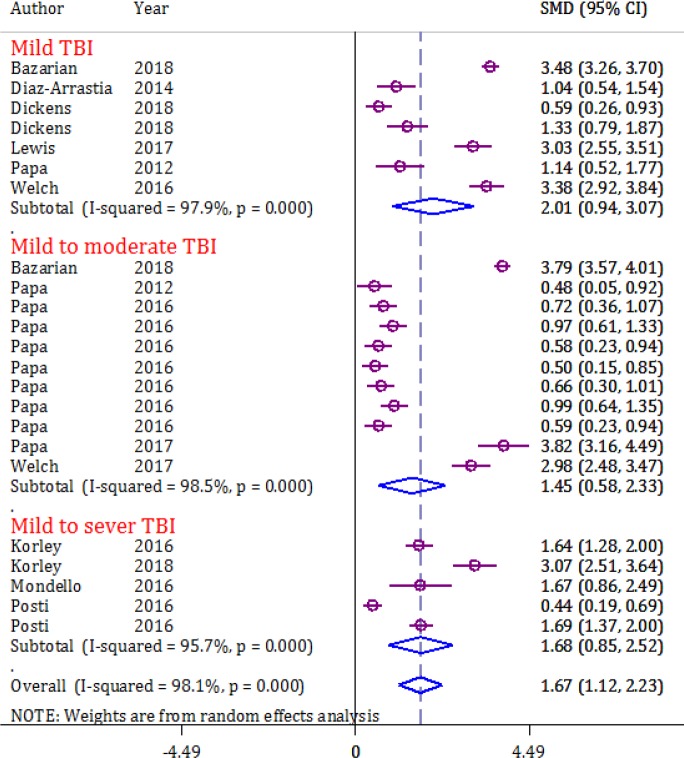
Forest plot for serum level of ubiquitin C-terminal hydrolase L1 in traumatic brain injury (TBI) subjects with positive computed tomography (CT) findings compared to negative CT findings. CI: Confidence interval; SMD: Standardized mean difference.

The mean value of serum/plasma levels of UCH-L1 reported in each of the 13 papers were investigated (8, 25-36). The analyzes showed that the mean serum/plasma level of UCH-L1 was significantly higher in CT-positive TBI than in CT negative TBI patients (SMD = 1.67, 95% CI: 1.12 to 2.23, p <0.0001; I2 = 98.1%, p < 0.0001) (Figure 3).

The mean serum/plasma level of UCH-L1 within 6 hours after TBI (SMD = 1.72, 95% CI: 0.98 to 2.47, p <0.0001), during the first 12 hours after injury (SMD = 1.74, 95% CI: 0.42 to 3.07, p = 0.01) and 24 hours later (SMD = 1.55, 95% CI: 0.88 to 2.21, p <0.0001) in CT positive TBI patients were always higher than CT negative patients.

**Screening performance characteristics in the detection of an intracranial lesion in mild TBI**

Six studies, including 15 separate experiments evaluated the performance of UCH-L1 in the prediction of intracranial lesions (8, 25, 26, 29, 30, 33). The cut off used in the studies varied between 41 pg/ml and 327 pg/ml. 11 experiments were performed on mild TBI, three experiments on mild to moderate TBI and one experiment on mild to severe TBI. Therefore, the analyzes of this section focused on mild TBI.

**Figure 4:**
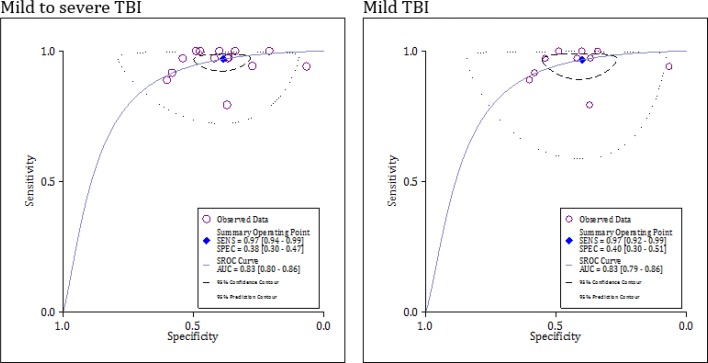
Summary of receiver operating curve (SROC) of serum level of ubiquitin C-terminal hydrolase L1 in detection of intracranial lesion (based on computed tomography findings) in mild and mild to severe traumatic brain injuries (TBI). AUC: Area under the curve; Sens: Sensitivity; Spec: Specificity.

**Figure 5:**
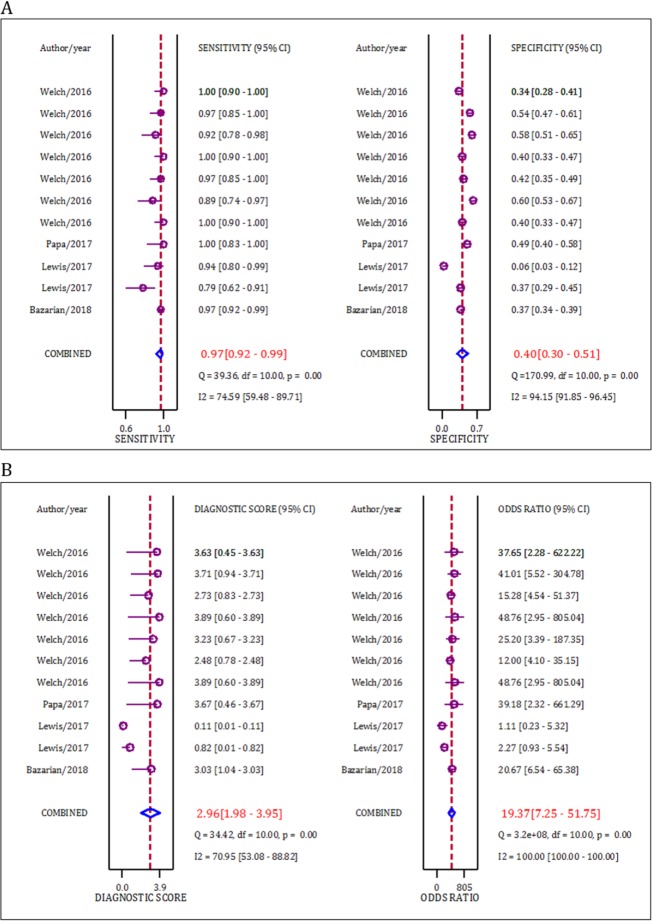
Performance of serum level of ubiquitin C-terminal hydrolase L1 in detection of intracranial lesion (based on computed tomography findings) in mild traumatic brain injuries.

The area under the SROC curve for serum/plasma UCH-L1 in the prediction of intracranial lesions after mild TBI was 0.83 (95% CI: 0.80 to 0.86) (Fig. 4). The sensitivity and specificity of this serum biomarker were 0.97 (95% CI: 0.92 to 0.99) and 0.40 (95% CI: 0.30 to 0.51), respectively. The Diagnostic odds ratio of UCH-L1 in the prediction of intracranial lesions after mild TBI was 19.37 (95% CI: 7.25 to 51.75) (Figure 5).

When the analysis was limited to assessing the serum/plasma UCH-L1 level within the first 6 hours after mild TBI, its sensitivity and specificity increased to 0.99 (95% CI: 0.94 to 1.0) and 0.44 (95% CI: 0.38 to 0.052), respectively. The diagnostic odds ratio of 6-hour UCH-L1 in the prediction of intracranial lesions was 680.87 (95% CI: 50.50 to 9197.97) (Table 3).

## Discussion:

The present meta-analysis showed that after TBI, the serum/plasma UCH-L1 level increased significantly. Therefore, it could be used as a biomarker to detect intracranial lesions. The area under the SROC of UCH-L1 in prediction of head CT scan findings was 0.83. Serum/plasma UCH-L1 has a high sensitivity (0.97) to predict intracranial lesions but its specificity (0.40) is low. Since the role of using biomarkers in the clinic is more focused on its screening value, the high sensitivity of UCH-L1 in predicting intracranial lesions is an advantage, while its low specificity is not a major weakness in the use of UCH-L1 in the management of TBI. However, it is necessary to introduce an optimum cut off for the serum/plasma level of UCH-L1.

The cut offs used in the studies were between 41 pg / ml and 327 pg / ml. Therefore, in the current meta-analysis, the assessment of the best cut offs for the UCH-L1 was not possible. Therefore, further studies are recommended.

The diagnostic/predictive value of biomarkers varies with time (37, 38). Since, decision making in TBI patients is performed during the first 24 hours of injury, the 24-hour serum/plasma UCH-L1 level was analyzed in the current meta-analysis. The findings indicate that the performance of UCH-L1 is higher in the first 6 hours of TBI than in the next few hours. Therefore, it seems that evaluating this biomarker as soon as possible can provide valuable information about the severity of TBI.

In a similar meta-analysis study, Shahjouei et al. showed that the serum/plasma level of UCH-L1 has a moderate value in the prediction of intracranial lesions (39). This report was different from the findings of the present study. Shahjouei et al., included data from four studies, while in the present study, data from six studies containing 15 separate experiments were entered. On the other hand, the conclusion presented by Shahjouei et al is based on the area under the curve of UCH-L1, while our findings were presented based on TP, TN. FP, and FN. In addition, the area under the curve simultaneously represents sensitivity and specificity. In the present study, the UCH-L1 has a high sensitivity that is very suitable for a screening test, but has a low specificity that does not play a critical role in screening of patients. Therefore, it is not possible to accurately discuss about the value of a screening biomarker just by reporting the AUC.

**Table 1:** Summary characteristics of studies

**Author; year; country**	**Type of study**	Age*	**CT- / CT+**	**Male gender**	**Sampling methods**	**Time to sample#**	**Method assay**	**GCS**
Bazarian; 2018; USA	Cohort	18-98	1793/122	1107	Convenience	0 to 12	ELISA	9-15
Diaz-Arrastia; 2014; USA	Cohort	37±14	40 / 31	150	NR	0 to 24	ELISA	14-15
Dickens; 2018; Finland and UK	Cohort	18-91	95 / 114	152	NR	0 to 12	ELISA	13-15
Korley; 2016; USA	Cohort	26-56	84 / 75	215	Convenience	0 to 24	ELISA	3-15
Korley; 2018; USA	Cohort	24-61	63 / 44	78	Convenience	0 to 24	ELISA	3-15
Lewis; 2017; USA	Trial	18-80	154 / 34	116	NR	0 to 24	ELISA	13-15
Mondello; 2016; USA	Cohort	3.8±3.7	10 / 29	28	NR	0 to 24	ELISA	3-15
Papa; 2012; USA	Cohort	18-89	77 / 28	64	Convenience	0 to 4	ELISA	9-15
Papa; 2016; USA	Cohort	18-83	290 / 35	212	Convenience	0 to 24	ELISA	9-15
Papa; 2017; USA	Cohort	0-21	134 / 17	100	Convenience	0 to 6	ELISA	9-15
Posti; 2016; Finland and UK	Cohort	45.3±19.2	90 / 200	239	Consecutive	0	ELISA	3-15
Welch; 2016; USA	Cohort	18-80	215 / 36	151	NR	0 to 6	ELISA	13-15
Welch; 2017; USA	Cohort	18-80	134 / 33	102	NR	0	ELISA	9-15

*, Data are presented as mean ± standard deviation or age range. #, hours from Traumatic brain injury (TBI). CT: Computed tomography; GCS: Glasgow coma scale of patients at admission; ELISA: Enzyme-linked immunosorbent assay; NR: Not reported.

**Table 2:** Quality assessment of included articles based on QUADAS-2 guideline

**Author; year**	**Risk of bias**					**Applicability**		
	**Patient selection**	**Index test**	**Reference standard**	**Flow and timing**		**Patient selection**	**Index test**	**Reference standard**
Bazarian; 2018	**◄**	**►**	**►**	**►**		**►**	**►**	**►**
Diaz-Arrastia; 2014	**?**	**►**	**►**	**►**		**►**	**►**	**►**
Dickens; 2018	**?**	**►**	**►**	**►**		**►**	**►**	**►**
Korley; 2016	**◄**	**►**	**►**	**►**		**►**	**►**	**►**
Korley; 2018	**◄**	**►**	**►**	**►**		**►**	**►**	**►**
Lewis; 2017	**?**	**►**	**►**	**►**		**►**	**►**	**►**
Mondello; 2016	**?**	**►**	**►**	**►**		**►**	**►**	**►**
Papa; 2012	**◄**	**►**	**►**	**►**		**►**	**►**	**►**
Papa; 2016	**◄**	**►**	**►**	**►**		**►**	**►**	**►**
Papa; 2017	**◄**	**►**	**►**	**►**		**►**	**►**	**►**
Posti; 2016		**►**	**►**	**►**		**►**	**►**	**►**
Welch; 2016	**?**	**►**	**►**	**►**		**►**	**►**	**►**
Welch; 2017	**?**	**►**	**►**	**►**		**►**	**►**	**►**

**►**: Low risk; **◄**: High risk; ?: Unclear

**Table 3:** Sensitivity analysis for performance of serum level of ubiquitin C-terminal hydrolase L1 in detection of intracranial lesion (based on computed tomography findings) in mild traumatic brain injuries

**Variable**	**Sensitivity**	**Specificity**	**Diagnostic score**	**Diagnostic odds ratio**
**Age group**				
Children	NA	NA	NA	NA
Adult	0.96 (0.92 to 0.98)	0.39 (0.29 to 0.51)	2.82 (1.86 to 3.77)	16.72 (6.44 to 43.39)
**Timing (hours after TBI)**				
0 to 6	0.99 (0.94 to 1.0)	0.44 (0.38 to 0.52)	6.52 (3.92 to 9.12)	680.87 (50.50 to 9197.97)
>6	NA	NA	NA	NA
**Overall**	0.97 (0.92 to 0.99)	0.40 (0.30 to 0.51)	2.96 (1.98 to 3.95)	19.37 (7.25 to 51.75)

All data are presented with 95% confidence interval.

Many articles do not report TP, TN, FP, and FN and instead provide sensitivity and specificity. To overcome this problem, using standard methods and web-based applications, TP, TN, FP, and FN ​​were obtained from sensitivity and specificity. This was one of the strengths of the present study. However, in the present study due to the high diversity among eligible studies, it was not possible to report the best cut off for serum/plasma level of UCH-L1. There was also a significant heterogeneity between studies. Unfortunately, we did not find the source of heterogeneity. Therefore, for these reasons, the evidence presented in this study was reduced to a moderate level.

## Conclusion:

A moderate level of evidence suggests that the serum/plasma level of UCH-L1 had good value in prediction of head CT findings. It was also found that evaluation of serum/plasma level of UCH-L1 within the first 6 hours after TBI would increase its predictive value. However, there is a controversy about the best cut offs of the UCH-L1 and further studies are needed.
